# Bolometric detection of Josephson radiation

**DOI:** 10.1038/s41565-024-01770-7

**Published:** 2024-08-22

**Authors:** Bayan Karimi, Gorm Ole Steffensen, Andrew P. Higginbotham, Charles M. Marcus, Alfredo Levy Yeyati, Jukka P. Pekola

**Affiliations:** 1 Pico Group, QTF Centre of Excellence, Department of Applied Physics, Aalto University, Espoo, Finland; 2 QTF Centre of Excellence, Department of Physics, Faculty of Science, University of Helsinki, Helsinki, Finland; 3 Departamento de Física Teórica de la Materia Condensada, Condensed Matter Physics Center (IFIMAC) and Instituto Nicolás Cabrera, Universidad Autonoma de Madrid, Madrid, Spain; 4 Instituto de Ciencia de Materiales de Madrid (ICMM), Consejo Superior de Investigaciones Científicas (CSIC), Madrid, Spain; 5 The James Franck Institute and Department of Physics, University of Chicago, Chicago, IL USA; 6 IST Austria, Klosterneuburg, Austria; 7 Materials Science and Engineering and Department of Physics, University of Washington, Seattle, WA USA; 8 Center for Quantum Devices, Niels Bohr Institute, University of Copenhagen, Copenhagen, Denmark; 9 InstituteQ—The Finnish Quantum Institute, Aalto University, Espoo, Finland

**Keywords:** Sensors, Superconducting devices

## Abstract

One of the most promising approaches towards large-scale quantum computation uses devices based on many Josephson junctions. Yet, even today, open questions regarding the single junction remain unsolved, such as the detailed understanding of the quantum phase transitions, the coupling of the Josephson junction to the environment or how to improve the coherence of a superconducting qubit. Here we design and build an engineered on-chip reservoir connected to a Josephson junction that acts as an efficient bolometer for detecting the Josephson radiation under non-equilibrium, that is, biased conditions. The bolometer converts the a.c. Josephson current at microwave frequencies up to about 100 GHz into a temperature rise measured by d.c. thermometry. A circuit model based on realistic parameter values captures both the current–voltage characteristics and the measured power quantitatively. The present experiment demonstrates an efficient, wide-band, thermal detection scheme of microwave photons and provides a sensitive detector of Josephson dynamics beyond the standard conductance measurements.

## Abstract (web summary)

An on-chip nano-bolometer integrated with a Josephson junction quantitatively measures the Josephson radiation up to about 100 GHz frequency. This wide-band, thermal detection scheme of microwave photons provides a sensitive detector of Josephson dynamics beyond the standard conductance measurements.

## Main

Understanding the dissipative dynamics of a Josephson junction (JJ)^1–6^ has been a topic of intensive studies ever since the seminal theoretical works of Ivanchenko and Zil’berman^7^ and of Caldeira and Leggett^8^. Nowadays, a JJ serves as a versatile component with a wide range of applications in various areas such as quantum computing and metrology^9^. Radiation from different types of JJ, including semiconductor nanowires and thin-film microbridges, has been detected by observing photon-assisted tunnelling, current–phase relationship and properties of resonant cavities^10–17^. The concept of our experiment—converting a.c. Josephson current at microwave frequencies to measurable d.c. power—is based on nano-bolometric techniques^18–25^. We use a hot-electron bolometer (HEB), which consists of a normal-metal nano-absorber whose temperature is measured by a normal-metal–insulator–superconductor (NIS) thermometer probe^26,27^. Owing to the quadratic response of the bolometer, simple d.c. measurement of the absorber temperature yields the magnitude of the Josephson current at frequencies up to about 100 GHz.

In the experiment, we simultaneously measure the average (charge) current through the junction and the HEB signal. The temperature of the HEB is able to resolve the transport characteristics of the JJ with high resolution, and it reveals complementary features compared with those given by the d.c. charge transport characteristics. We find that a simple lumped-element description of the local environment accounts quantitatively for both the temperature and the charge transport characteristics, with the ubiquitous drop in the measurements stemming from a circuit resonance.

**Fig. 1:**
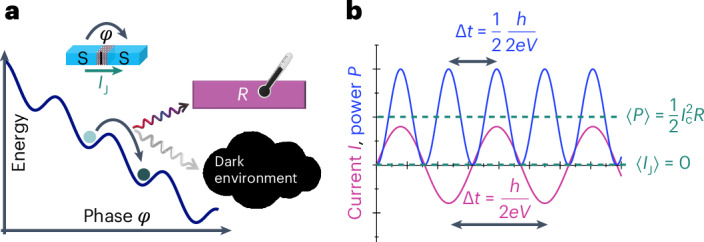
Energy released from a biased SIS JJ. **a**, The conceptual illustration of the energy versus the phase drop *φ* across the biased JJ. This junction emits energy either to the engineered absorber on the chip *R* or to the dark environment. **b**, The time average current 〈*I*_J_〉 = 0 vanishes ideally in a voltage-biased junction, whereas the average power remains non-vanishing.

The conventional picture of a current-biased JJ is that of a tilted washboard potential against the phase bias *φ* across it as in Fig. 1a, where the slope is given by the bias. At small currents, the phase particle remains trapped in one of the wells of this potential, and since the voltage *V* is proportional to the time derivative of this phase, given by the Josephson relation as $V=\frac{\hslash }{2e}{\rm{d}}\varphi /{\rm{d}}t$, the junction remains in its zero-voltage supercurrent state. Here *ℏ* is the reduced Planck’s constant and *e* is the electron charge. The current in the junction is thus dissipationless. Upon increasing the tilt of the potential, the phase starts to move from one well to the next lower one, producing a non-vanishing voltage; that is, the process becomes dissipative. Each jump in the phase from one well to the next lower one releases energy equal to Φ_0_*I*, where Φ_0_ = *h*/2*e* is the superconducting flux quantum and *I* is the average current. The current experiment deals, however, mainly with a voltage-biased configuration where the picture is less understood.

The current through the junction at non-vanishing voltage is given by the Josephson relation, ${I}_{{\rm{J}}}={I}_{\rm{c}}\sin \varphi$, where *I*_c_ is the junction-specific critical current, and the phase is evolving according to *φ*(*t*) = 2*eVt*/*ℏ*. The mean-squared value of this current is then given by ${I}_{\rm{c}}^{2}/2$. The conceptual difference between the measurement of current and power is illustrated in Fig. 1b. A key question is, can this alternating a.c. Josephson current be fully detected by the bolometer operating in d.c. mode; that is, can we measure the power equal to $P={I}_{\rm{c}}^{2}R/2$ via a temperature measurement of the resistor with resistance *R*? We will first introduce the experimental set-up and results, which allow us to answer this question affirmatively. The second key question in our work is, where does this energy get absorbed? Can it be collected by a bolometric detector or is it released to an unknown environment that cannot be monitored?

## Experimental set-up

**Fig. 2:**
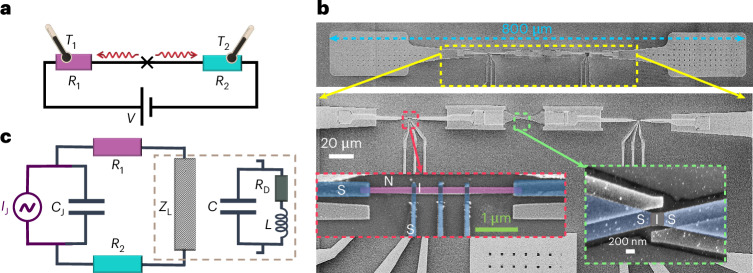
A set-up consisting of the JJ surrounded by two bolometers. **a**, A schematic illustration of the device where the energy from the voltage *V*-biased JJ (cross-shaped structure) at angular frequency *ω*_J_ = 2*eV*_JJ_/*ℏ* is emitted to the two absorbers with resistances *R*_1_ and *R*_2_. Here *V*_JJ_ ≃ *V* is the voltage drop across the JJ. The thermometers on the absorbers measure the temperature changes of them, *T*_1_ and *T*_2_, simultaneously. **b**, A scanning electron micrograph of the device on different scales. A single JJ in the middle is sandwiched between two resistors placed at a distance of about 100 μm from the JJ. The connections are made with aluminium (Al) and with niobium (Nb, the wider sections). The zoomed-in views highlight the SIS junction (Al, blue; AlO_*x*_, grey) in the middle and the left side, one of the absorbers (resistor *R*_1_) made of copper in purple colour, in clean contact with Al (blue) leads connecting to the patterned niobium film (light grey). The temperature of the absorber is monitored and controlled by three NIS probes (Cu/AlO_*x*_/Al). **c**, Lumped-element model of the device for radio-frequencies; a single JJ represented by a current source *I*_J_, an ideal Josephson element, and capacitance *C*_J_ in parallel. The load impedance *Z*_L_ is the termination of the device via bonding pads and wire bonds, and it is modelled as a dissipative *LCR*_D_ element as shown. Here *R*_D_ is the resistor representing the dark environment. Source data

A schematic representation of the set-up is shown in Fig. 2a where the energy is released by the JJ biased at voltage *V*, indicated by the cross. As will be clear in what follows, the bias voltage *V* is dropping almost fully across the junction (*V* ≃ *V*_JJ_), except in the trivial supercurrent branch (*V*_JJ_ = 0.0). The temperatures *T*_1_ and *T*_2_ of the absorbers are measured by the thermometers attached to them. Figure 2b shows the actual device, where a single JJ (0.3 × 0.3 μm^2^) in the middle is positioned between two normal-metal resistors, *R*_1_ and *R*_2_, made of copper (2.5 × 0.1 × 0.03 μm^3^), working as absorbers, at a distance of about 100 μm symmetrically around the JJ. The connections are made of aluminium whose thermal conductance is negligible at low temperatures^28,29^. Larger features, namely, contact leads, bonding pads and the surrounding area (light grey in Fig. 2b), are made of niobium. By current biasing a pair of normal-metal–insulator–superconductor junctions (SINIS) attached by superconducting leads to the resistors, one can control and monitor their temperatures. The voltage across the junction at a fixed bias current measured at different bath temperatures yields the temperature calibration as presented in Supplementary Figs. 3 and 4 with details explained. The resistance of each resistor is about 15 Ω, obtained by four-probe measurements at low temperature. The simplified equivalent circuit of the device, used in the theoretical modelling of the *I*–*V* characteristics and power, is presented in Fig. 2c. Here a single JJ is represented by an a.c. current source in parallel with capacitance *C*_J_, and *Z*_L_ is the external impedance formed mainly by the bonding pads and wires.

## Charge transport in different regimes

**Fig. 3:**
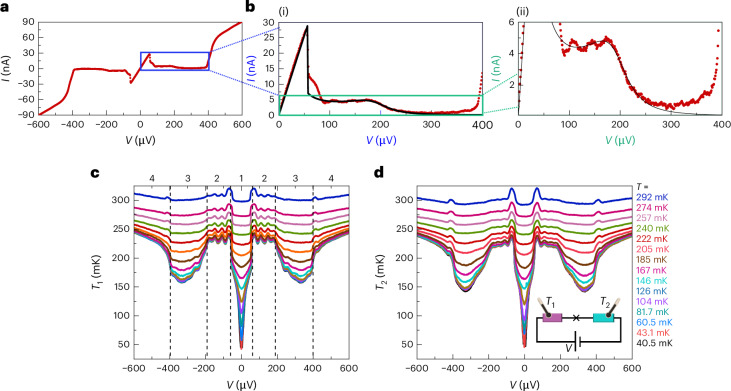
Transport characteristics of the device of Fig. 2b. **a**, The *I*–*V* characteristic shown with red symbols measured at *T*_0_ = 50 mK. **b**(i),(ii), Zoom-in of the enclosed area in blue shown in **a**. The black solid line is from the theoretical model with parameters: *R*_1_ + *R*_2_ = 30 Ω, *R*_D_ = 120 Ω, *R*_S_ = 1,920 Ω, *C*_J_ = 2.4 fF, *C* = 6.5 fF, *L* = 0.34 nH and *I*_c_ = 64 nA. **c**,**d**, Simultaneous measurement of the temperature of the resistor *T*_1_ in panel **c** and *T*_2_ in panel **d** as a function of applied bias *V*. Source data

**Extended Data Fig. 1:**
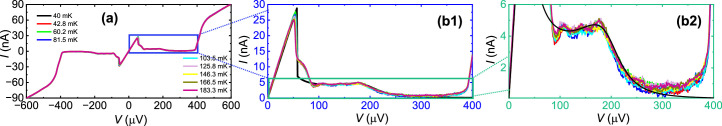
The *I* − *V* characteristics measurement. (**a**) The *I* − *V* measured at different bath temperatures, *T*_0_ = 40 − 183 mK. (**b1**),(**b2**) Zoom-in of the enclosed area in blue shown in panel (**a**). Source data

The *I*–*V* characteristic of the device at *T*_0_ = 50 mK is presented in Fig. 3a (the measurement of *I*–*V*’s at different temperatures is shown in Extended Data Fig. 1). In the supercurrent branch in the central part (see Supplementary Section IV for the value of the switching current *I*_s_), the positive constant slope is a result of the series resistance *R*_S_ from the two absorbers, the load resistors on the chip, and line and bias resistances from room temperature to the chip (*R*_S_ ≈ 1,920 Ω) as we plot the current *I* versus the applied voltage *V*. The almost flat section up to the gap, *V* = 2Δ/*e* ≃ 400 μV, is followed by a rise in current, transitioning into a quasiparticle current with specific resistance of the JJ, *R*_T_ = 5.0 kΩ. The magnified view of the enclosed area of the *I*–*V* characteristic in panel a, as shown in Fig. 3b(i),(ii), provides a closer look at this measurement. It unveils additional details, particularly the oscillations between ∣*V*∣ = 50–200 μV and drop in current at ∣*V*∣ = Δ/*e* ≃ 200 μV in the subgap regime. We assume that the gap has its zero temperature value as we work at temperatures <0.3*T*_c_, where *T*_c_ ≃ 1.3 K is the critical temperature of aluminium.

We now analyse theoretically the *I*–*V* profile in the almost flat subgap region, 50 μV ≲ *V* ≲ 400 μV, in terms of an underdamped JJ in series with a frequency-dependent impedance *Z*(*ω*). In this regime, the phase evolves as

1 $$\varphi (t)\approx {\omega }_{{\rm{J}}}t+\frac{2e}{\hslash }\frac{{I}_{\rm{c}}| Z({\omega }_{{\rm{J}}})| }{{\omega }_{{\rm{J}}}}\sin \left({\omega }_{{\rm{J}}}t+\delta \right)$$

with the phase shift $\delta =-\arctan (\,\text{Re}\,Z({\omega }_{{\rm{J}}})/\,\text{Im}\,Z({\omega }_{{\rm{J}}}))$. To the lowest order in voltage variations across the junction, the JJ d.c. current *I* ≡ 〈*I*_J_〉 and voltage drop are given by

2 $$I={I}_{\rm{c}}^{2}\frac{e}{\hslash }\frac{\,\text{Re}\,Z({\omega }_{\rm{J}})}{{\omega }_{\rm{J}}},\,\,V=\frac{\hslash }{2e}{\omega }_{{\rm{J}}}+Z(0)I,$$

with ${\omega }_{\rm{J}}=\frac{2e}{\hslash }{V}_{\rm{JJ}}$ denoting the emitted angular frequency. These equations are then solved to yield the plotted *I*–*V*. Intuitively, the proportionality of *I* on *Z*(*ω*_J_) arises from the dynamics of the phase particle in the washboard potential. The lower the friction, given by *Z*(*ω*_J_), more uniformly the phase runs down the washboard, resulting ideally in vanishing $I={I}_{\rm{c}}{\langle \sin \varphi (t)\rangle }_{0}$, while larger friction results in a more rugged motion of it, thereby increasing *I*. Here the subscript 0 denotes the average over the period. The *I*–*V* seen in Fig. 3b is reproduced by using the impedance of the a.c. circuit with resonance at *ω*_LC_/2*π* ≈ 100 GHz (LC refers to the tank circuit formed of the inductor and the total capacitance in the circuit in parallel). More details on the theory can be found in Supplementary Section V. This model captures the main features of the experimental *I*–*V* curve with realistic circuit parameters as shown by black lines in Fig. 3b(i),(ii).

## Bolometric detection of current

**Extended Data Fig. 2:**
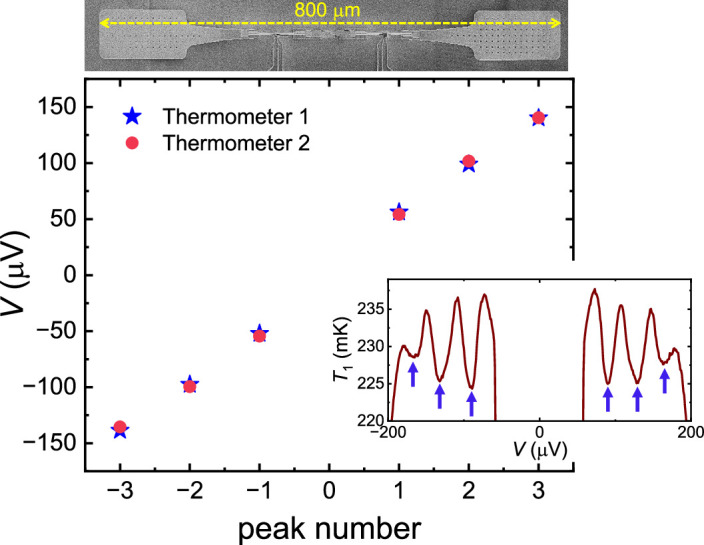
Temperature resonances in the JJ cavity. Extracted voltage bias points *V* versus the peak number of both bolometers presented in Fig. 3(c),(d). The inset shows a magnified view of the bias dependent temperature of *R*_1_ at base temperature *T*_0_ = 43 mK. The arrows point to the minima in temperature that have been extracted to the main panel. Source data

**Extended Data Fig. 3:**
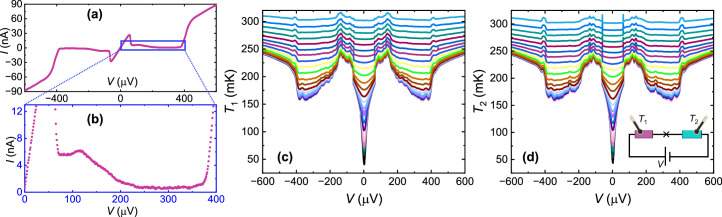
Similar data as in Fig. 3, but here measured on another sample with nominally same parameters as those of the sample in the main text but with different wire-bonding conditions. **a**, The *I*–*V* characteristic shown with symbols measured at *T*_0_ = 50 mK. **b**, Zoom-in of the enclosed area in blue shown in **a**. **c**, Measurement of the temperature *T*_1_ of *R*_1_ as a function of applied bias *V*. **d**, Same as **c** for *T*_2_ of *R*_2_. Source data

The energy released from the JJ at the given voltage bias is absorbed partly or fully by the two resistors. The measurement of temperatures *T*_1_ and *T*_2_ as a function of applied bias *V* is presented in Fig. 3c,d, respectively (see also additional data for another sample in Extended Data Fig. 3). The temperature calibration is explained in Supplementary Section III. The non-monotonic behaviour of temperatures is remarkably similar in the two absorbers. The temperature changes clearly indicate four different regimes based on the different sections of the *I*–*V* characteristic presented in Fig. 3a,b. These regimes are summarized in Table 1. In the central region, Regime 1 up to ∣*V*∣ ≃ 50 μV, the entire heating of the resistors is attributed to Joule heating, a consequence of the current flowing through the resistors, as there is no power dissipation within the JJ itself. The most interesting regime, Regime 2, lies within the intermediate range, 50 μV < ∣*V*∣ < 200 μV, where the energy released by the a.c. Josephson current at the frequency *f* = 2*eV*/*h* is absorbed by the resistors. In this regime, the temperature is high and shows small oscillations as a function of *I*, as discussed below and in the additional data of Extended Data Fig. 2. In Regime 3, 200 μV < ∣*V*∣ < 400 μV, the power emitted into the two resistors decreases, corresponding to reduction in their temperatures. At high voltage bias ∣*V*∣ > 400 μV, Regime 4, in the quasiparticle branch, only a small fraction of power generated within the junction is dissipated into the two absorbers, leading to a continuous, monotonous increase in their temperatures. Note that power generated at the junction in Regime 4, based on the d.c. *I*–*V*, is indeed more than 100 times higher than in Regime 2, but the temperature rise is comparable, indicating a different mechanism of heat transport in each regime.

**Table 1:** Different bias regimes

Regime	Range of ∣*V*∣	Process
Regime 1	<50 μV	Supercurrent
Regime 2	50–200 μV	Josephson radiation I *ω* < *ω*_LC_
Regime 3	200–400 μV	Josephson radiation II *ω* > *ω*_LC_
Regime 4	>400 μV	Quasiparticle current

The oscillations in Regime 2, spaced by about Δ*V* = 50 μV, seen in Fig. 3c,d and Extended Data Fig. 2, can be qualitatively understood as follows. The wavelength *λ* = *hc*/(2*eV*) yields *λ* = 4 mm for *V* = 50 μV; thus, the positions correspond to multiples of *λ*/4 as the whole device is about 800 μm long as shown in the top of Fig. 2b. It acts as a cavity with imperfect termination. This termination by bonding pads and inductive bonding wires causes partial reflection of current, leading to temperature variations in the absorbers visible in the subgap regime. The inset of the additional data in Extended Data Fig. 2 shows the temperature variation of the absorber 1 within the subgap regime. The blue arrows point to the minima of the measured temperature. The main panel of this figure shows the positions of the minima, which are the same for the two absorbers over the whole voltage range. Data on another sample are presented for comparison in Extended Data Fig. 3.

## Energy release by Josephson current

Our qualitative interpretation of the overall temperature characteristics in Fig. 3c,d and in the inset of Extended Data Fig. 2 is as follows. Beyond the pure Joule heating of the resistors by the d.c. current in Regime 1, the high level of heating in Regime 2 is attributed to the absorption of the a.c.-supercurrent-induced high-frequency (2*eV*/*h*) power in the corresponding resistor. Note that the d.c. current in Regime 2 is less than 5 nA. Thus, the Joule heating in the metallic resistor by this current is then less than (5 nA)^2^ × 15 Ω = 0.38 fW, which is 1% of the measured power by the a.c. Josephson current. As shown below, this d.c. power is very close to the average value ${P}_{i}=\frac{{I}_{\rm{c}}^{2}}{2}{R}_{i}$ for *i* = 1, 2. Crossover to Regime 3 is characterized by an abrupt decrease of power for both resistors. This drop has been seen in all the samples that we have measured, and it can be accounted by the drop in the current *I*: the circuit re-routes the current away from the thermometers at higher frequencies.

To understand this mechanism of re-routing, we note that based on our circuit model (Fig. 2c), the power deposited in the HEBs in the underdamped regime is given by

3 $${P}_{i}={R}_{i}{I}^{2}+\frac{{R}_{i}{I}_{\rm{c}}^{2}}{2}\left[{\left(1+{C}_{{\rm{J}}}{\omega }_{{\rm{J}}}\text{Im}Z({\omega }_{{\rm{J}}})\right)}^{2}+{\left({C}_{{\rm{J}}}{\omega }_{{\rm{J}}}\text{Re}Z({\omega }_{{\rm{J}}})\right)}^{2}\right],$$

which for small biases results in ${P}_{i}\approx \frac{{R}_{i}{I}_{\rm{c}}^{2}}{2}$, but for biases $V\gg \frac{\hslash }{2e}{\omega }_{\rm{LC}}$, the power approaches ${P}_{i}\approx \frac{{R}_{i}{I}_{\rm{c}}^{2}}{2}\frac{1}{{(1+{C}_{{\rm{J}}}/C)}^{2}}$. This is a consequence of the a.c. current bypassing the HEBs by running through *C* and *C*_J_, instead of *L*, at large frequencies. Regime 4 represents pure quasiparticle current in tunnelling, where the heat is released to the electrodes right at the junction in the form of hot quasiparticles. These non-equilibrium quasiparticles diffuse poorly in the superconductor and do not carry heat to the resistors as effectively as Josephson radiation does.

**Fig. 4:**
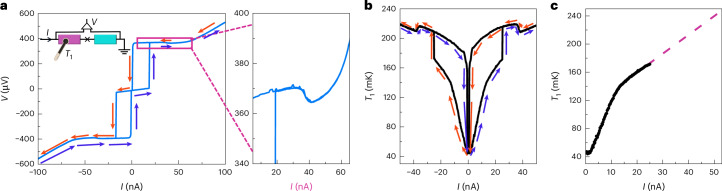
Characteristics of the current-biased JJ. **a**, Hysteretic *V*–*I* characteristics of the junction together with a zoom in the quasiparticle branch. The blue arrows show the signal in the forward current sweep, while the red ones present the reverse sweep. The set-up for voltage and temperature measurement is shown in the inset. **b**, Measured resistor temperature at current bias of *I*_1,th_ = 15 pA at *T*_0_ = 43 mK. The nearly parabolic bottom in the small bias range corresponds to the hysteretic supercurrent branch of the single JJ. **c**, The data in the supercurrent branch presented in **b** at positive currents. The dashed line indicates the linear extrapolation in the regime not reachable by the supercurrent measurement. Source data

The hysteretic measurement in the current-biased configuration, presented in Fig. 4a, gives us a way to perform the power calibration for the HEB. The key to this calibration is the measurement of the rise of the temperatures of the resistors *R*_*i*_ in the supercurrent branch of the JJ, where there is no dissipation in the junction, and all the power is the plain Joule heating by the d.c. current through the corresponding resistor. The data from one of them as a function of current *I* are shown at *T*_0_ = 43 mK in Fig. 4b. The blue arrows mark the current sweep in the forward direction, whereas the red ones denote the reverse direction. The hysteretic range at low currents corresponds to the non-dissipative supercurrent branch of the single JJ. Interestingly, we also see a drop in temperature at about 35 nA in the non-hysteretic quasiparticle branch. The *V*–*I* shown in Fig. 4b and its inset shows a drop at the same value of current, consistent with reduced heating above 35 nA. This drop could be associated with the ‘back-bending’ behaviour that has been observed in superconducting tunnel junctions^30,31^ owing to a suppression of the gap caused by the non-equilibrium distribution of the tunnelling quasiparticles.

**Fig. 5:**
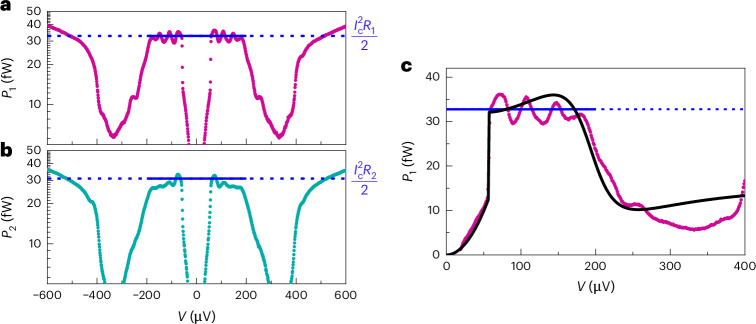
Bolometric detection of power radiated by the biased JJ. The power to the two bolometers based on temperature data in Fig. 3c,d. **a**, *P*_1_ as a function of the applied voltage *V*, obtained from the temperature measurement *T*_1_ of *R*_1_ at *T*_0_ = 43 mK. **b**, Same as **a** but *P*_2_ for *T*_2_ of *R*_2_. The conversion from *T* to *I* is obtained from the calibration presented in Fig. 4c. The horizontal blue line in each panel corresponds to ${I}_{\rm{c}}^{2}{R}_{i}/2$. **c**, In this panel, we have included the theoretical model, equation (3), to **a** using the same parameter values as for the *I*–*V* characteristic measurement presented in Fig. 3. Source data

In Fig. 4c, we present the measurement of temperature for positive currents only. To reach the temperatures in all the relevant ranges, we perform a linear extrapolation. Now having the relation between *I* and *T*_*i*_, we can convert each measured temperature *T*_*i*_ to effective power also in the voltage-biased configuration. The above calibration allows us to convert temperature plots presented in Fig. 3c,d to power injected to the resistor, *P*_*i*_ = *I*^2^*R*_*i*_ for *i* = 1, 2, as a function of bias voltage as presented in Fig. 5a,b. The maximum power injected in the subgap regime is about 35 fW in each resistor. This power corresponds to about 20% of *IV* at low bias voltages.

The single JJ in the voltage-biased configuration produces an a.c. Josephson current. We may compare the measured power with that expected if the a.c. Josephson current would produce Joule heating of resistor *i* at the rate ${P}_{i}={I}_{\rm{c}}^{2}{R}_{i}/2$, stipulated by the condition that all this current would pass through this purely resistive absorber. The copper wire can indeed be considered as a resistive element as its inductance and the capacitance across are small enough at the relevant microwave frequencies (<100 GHz). The critical current can be obtained from the Ambegaokar–Baratoff formula^32^, *I*_c_ = *π*Δ/(2*eR*_T_), applicable at temperatures far below the critical temperature *T*_c_ ≈ 1.3 K as in our experiment. The junction parameters were determined from the *I*–*V* curve of the JJ, with values Δ/*e* = 210 μV and *R*_T_ = 5.0 kΩ, yielding *I*_c_ = 64 nA. With this procedure, we obtain the expected powers *P*_1_ = 37 fW and *P*_2_ = 35 fW, indicated by the blue horizontal lines in Fig. 5, in good agreement with the maximum subgap power in the experiment. The result of the theoretical model is shown by the solid black line in Fig. 5c. Using the same parameter values as in Fig. 3, the full theoretical model captures the main features of the bias dependence in the experiment.

## Conclusion

In this article, we have demonstrated experimentally bolometric detection of a.c. Josephson current. It is a complementary d.c. method with respect to measuring the *I*–*V* characteristics: the standard *I*–*V* or conductance measurement essentially averages the sinusoidal current, whereas the HEB measurement is quadratic in current, thus rectifying and fully retaining the a.c. component. This way, still by a simple d.c. measurement, we gain access to a.c. characteristics, which are in general averaged out, thus challenging to measure in standard transport measurements. We presented a theoretical model that agrees with the experimental results using realistic circuit parameter values. It also gives a way to estimate the value of *R*_D_, which presents the unknown dark environment. Our model also shows that one could improve the detection efficiency of the HEB by increasing its resistance as this quantity scales approximately as (*R*_1_ + *R*_2_)/(*R*_D_ + *R*_1_ + *R*_2_).

## Methods

### Fabrication

Our process consists of three steps: fabricating (i) the Nb patterned ground planes, (ii) single superconductor–insulator–superconductor (SIS) JJ and (iii) absorbers and thermometers. The devices were fabricated on highly resistive 675 μm thick silicon substrates onto which a 40 nm aluminium-oxide layer by atomic layer deposition has been grown. Next, it is coated with a 200-nm-thick sputtered niobium film. Broader features such as contact leads and bonding pads were patterned by reactive ion etching using CF6 + Ar chemistry on an electron-beam lithography-defined mask. The JJ and NIS tunnel junctions were fabricated using a two-step process. First, shadow-mask electron-beam lithography was used to pattern them onto a 950-nm-thick poly(methyl methacrylate)/copolymer resist bilayer. This step was followed by developing the exposed structures in methyl-isobutyl-ketone in isopropanol developer and methylglycol-methanol solution for making undercuts, respectively.

Next, the junctions were deposited using the standard Dolan bridge technique by an electron-beam evaporator. Before metal evaporations, in situ argon plasma milling was conducted on the sample surface. This milling process aimed to remove the native oxide layer, providing a pristine interface between the Nb and Al layers. In the first step, a two-angle deposition process of 30-nm-thick Al layers with intermediate in situ oxidation is performed to realize the single JJ with a tunnel resistance of 5 kΩ at mK temperatures.

The bolometers are made in the second step as shown in Fig. 2, similar to the preceding step. The procedure initiates with in situ Ar ion plasma milling to ensure a clean contact between Nb and the deposited metals. The procedure then continues by first depositing a 20-nm-thick Al layer and in situ oxidation, followed by a 30-nm-thick Cu layer, and finally by a 50-nm-thick Al layer in clean contact with the Cu layer. The typical tunnel junction resistance for NIS for this experiment is about 20 kΩ. These NIS electrodes (probes) are connected to bonding pads for the setting and readout of the electronic temperature of the absorbers (*R*_1_ and *R*_2_).

The final stage of the fabrication is lift-off in acetone (52 degrees for ~20–30 min) and cleaning in isopropyl alcohol. After the fabrication process, the device is bonded with Al wires to a custom-made brass chip carrier for the cryogenic characterization.

## Online content

Any methods, additional references, Nature Portfolio reporting summaries, source data, extended data, supplementary information, acknowledgements, peer review information; details of author contributions and competing interests; and statements of data and code availability are available at 10.1038/s41565-024-01770-7.

## Supplementary information

Supplementary Information Supplementary Figs. 1–7 and Discussion.

## Source data

Source Data Fig. 2 Original SEM images of the measured device, with no colour and full size.

Source Data Fig. 3 Source data for Fig. 3. Panels separated in different sheets.

Source Data Fig. 4 Source data for Fig. 4. Panels separated in different sheets.

Source Data Fig. 5 Source data for Fig. 5. Panels separated in different sheets including the numerical values from theory.

Source Data Extended Data Fig./Table 1 Source data for Extended Data Fig. 1. Panels separated in different sheets including the numerical values from theory.

Source Data Extended Data Fig./Table 2 Source data for Extended Data Fig. 2. Panel and the inset data separated in different sheets.

Source Data Extended Data Fig./Table 3 Source data for Extended Data Fig. 3. Panels separated in different sheets.

## Data Availability

All data will be made available online, 10.5281/zenodo.12684006. Source data are provided with this paper.

## Code availability

All codes for reproducing the theory curves in the paper will be made available online, 10.5281/zenodo.12684006.
