# Supplementary material for: Bolometric detection of Josephson radiation
Source: Nat Nanotechnol. 2024 Aug 22;19(11):1613–8. doi: 10.1038/s41565-024-01770-7 (PMC11567893; doi:10.1038/s41565-024-01770-7)
Supplement: Supplementary file 1 — Supplementary Figs. 1–7 and Discussion. [file 41565_2024_1770_MOESM1_ESM.pdf]

nature nanotechnology

Supplementary information https://doi.org/10.1038/s41565-024-01770-7

Bolometric detection of Josephson radiation

In the format provided by the authors and unedited

(Dated: July 10, 2024)

CONTENTS

I. Chip design 2

II. Measurement configuration 2

III. Temperature calibration 2

IV. Switching current I_s versus critical current I_c 2

V. Theory and fitting details 4

References 7

I. CHIP DESIGN

Figure S1 presents the global view of the measured device. The light areas represent metallic Nb film.

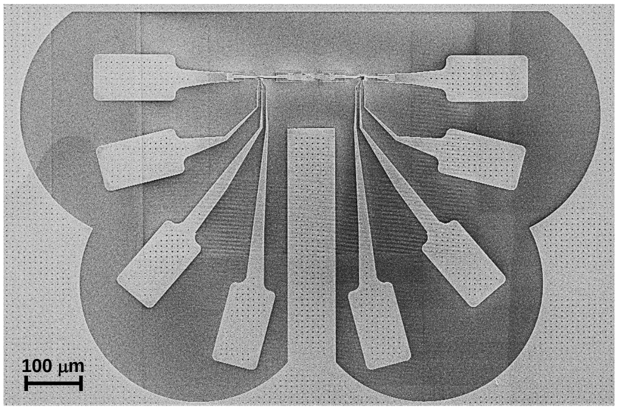

FIG. S1. The overall structure of the sample being studied.

II. MEASUREMENT CONFIGURATION

Figure S2 illustrates the complete measurement configuration utilized in this study. In order to mitigate potential adverse influence of the virtual grounding and impedance in the current amplifier, we opted to perform the current measurement via the voltage drop across a fixed resistor.

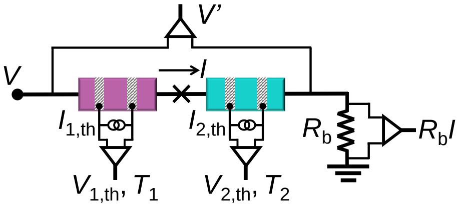

FIG. S2. The complete measurement configuration.

III. TEMPERATURE CALIBRATION

A pair of NIS-junctions attached to each resistor R_i serves as a thermometer. We apply a constant current $I_{\rm th,i}$ through this pair and measure the voltage across it [4], see Figs. S3 and S4. The calibration is done under equilibrium when the JJ bias current (and voltage) are set to zero. Under these conditions we assume that the electronic temperature of R_i equals that of the cryostat T_0, measured independently using a calibrated $\rm RuO_x$ thermometer. Figures S3 and S4 are examples of the calibrations for the data in the main text.

IV. SWITCHING CURRENT I_s VERSUS CRITICAL CURRENT I_c

It is well known that the switching current I_s of a small Josephson junction is much smaller than the (Ambegaokar-Baratoff) critical current I_c. For instance in experiments in [1] this suppression was as large as 70%, and depended strongly on the value of I_c. According to Refs. [1, 2], the escape rate for the macroscopic quantum tunneling (MQT) is given by

$$\Gamma_{\rm MQT} = \alpha\, e^{-\frac{36}{5}\frac{\Delta U}{\hbar\omega_p}}, \tag{1}$$

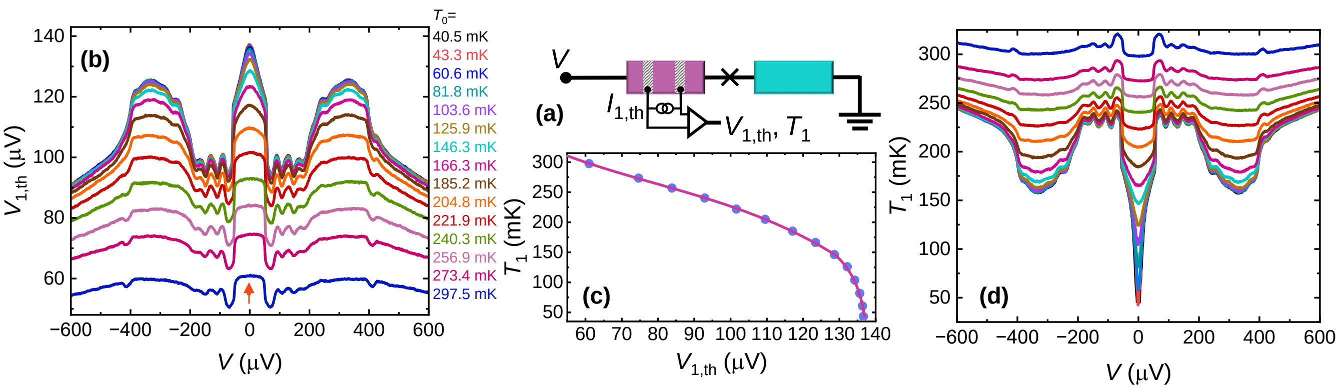

FIG. S3. Temperature calibration of the voltage-biased JJ. (a) Voltage-biased measurement setup. (b) The thermometer voltage $V_{1,\text{th}}$ is measured at a fixed current $I_{1,\text{th}} = 15\,\text{pA}$ as shown in (a) at different bath temperatures $T_0 = 43 - 298\,\text{mK}$ from top to bottom, against the voltage V across the JJ. (c) The equilibrium values of $V_{1,\text{th}}$ presented by symbols, at $V = 0$ (the position is shown by the red arrow in panel (b), serve as the calibration $V_{1,\text{th}}(V = 0)$, versus T_1. Solid line is fit to the medasured data. At $40\,\text{mK} \leq T_1 < 145\,\text{mK}$, the fitting function is $T_1 = a + b * \lg(c - V_{1,\text{th}})$ with a, b and c as constant, and at $145\,\text{mK} \leq T_1 < 350\,\text{mK}$ we used standard third-order polynomial (nearly linear) function. (d) Temperature of the resistor R_1 as a function of applied voltage at different bath temperatures T_0.

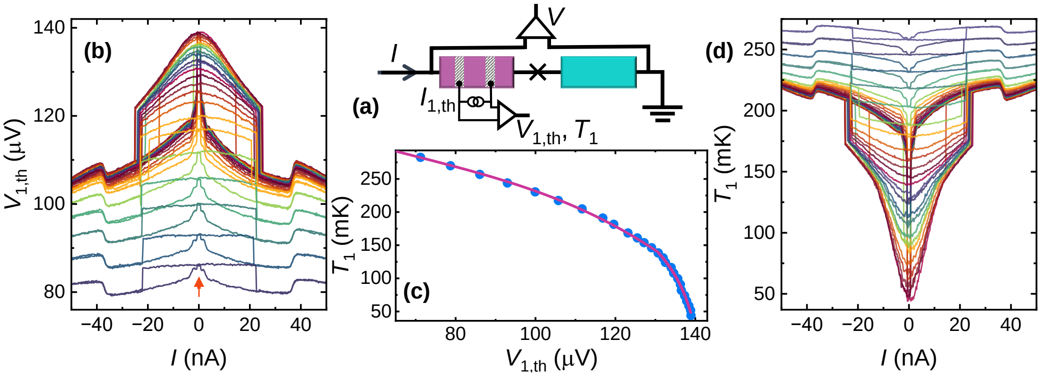

FIG. S4. Temperature calibration of the current-biased JJ. (a) Current-biased measurement setup. (b) The thermometer voltage $V_{1,\text{th}}$ is measured at a fixed current $I_{1,\text{th}} = 15\,\text{pA}$ as shown in (a) at different bath temperatures $T_0 = 43 - 256\,\text{mK}$ from top to bottom, against the current through JJ. (c) The equilibrium values of $V_{1,\text{th}}$ (symbols), at $I = 0$ (the position is shown by the red arrow in panel (b), serve as the calibration $V_{1,\text{th}}(I = 0)$, versus T_1. Solid line presenting the fit to the experimental data is similar to the one in Fig. S3(c) meaning $T_1 = a + b * \lg(c - V_{1,\text{th}})$ with a, b and c as constant and third-order polynomial function in the corresponding T_1 ranges. (d) Temperature of the resistor R_1 as a function of applied current at different bath temperatures T_0. Note that the current dependence in (b) and as a result in (d) is hysteretic in I like in Fig. 4(b) in the main text.

where $\alpha = 12\sqrt{6\pi}\frac{\omega_p}{2\pi}\sqrt{\frac{\Delta U}{\hbar\omega_p}}$, $\omega_p = \sqrt{8\,E_J\,E_C}/\hbar$ is the plasma angular frequency and ΔU denotes the barrier height in the washboard potential, and for the cubic approximation is given by $\Delta U = \frac{2}{3}E_J\,[2(1 - I/I_c)]^{3/2}$ at current I. Knowing that below the cross-over temperature $T_0 = \hbar\omega_p/(2\pi k_B) \simeq 300\,\text{mK}$, the dominant escape mechanism is MQT, the escape probability in the time interval $0 \leqslant t \leqslant \tau$ is given by $P = 1 - e^{-\int_0^\tau \Gamma_{\text{MQT}}(I)dt}$ at a fixed current. In order to obtain the escape current (switching current) in this case, it is enough to calculate I/I_c when $\Gamma_{\text{MQT}}(I)\tau \approx 1$. Using Eq. (1) and after a little algebra we have

$$\frac{I_s}{I_c} \approx 1 - \left[\ln(\alpha\tau)\frac{5}{24}\sqrt{\frac{E_C}{E_J}}\,\right]^{\frac{2}{3}}. \qquad (2)$$

Based on the critical current given in the main text $I_c = 64.4\,\text{nA}$, we have $E_J/k_B = 1.54\,\text{K}$. To find a good approximation for E_C, we measured the conductance of a long array of similar Josephson junctions (nominally identical to the present one and in the normal state) in the weak Coloumb blockade regime [3]; we obtain $E_C/k_B = 0.3\,\text{K}$. This value is consistent with the expectation based on the area of the junction using $50\,\text{fF}/(\mu\text{m})^2$ as specific junction

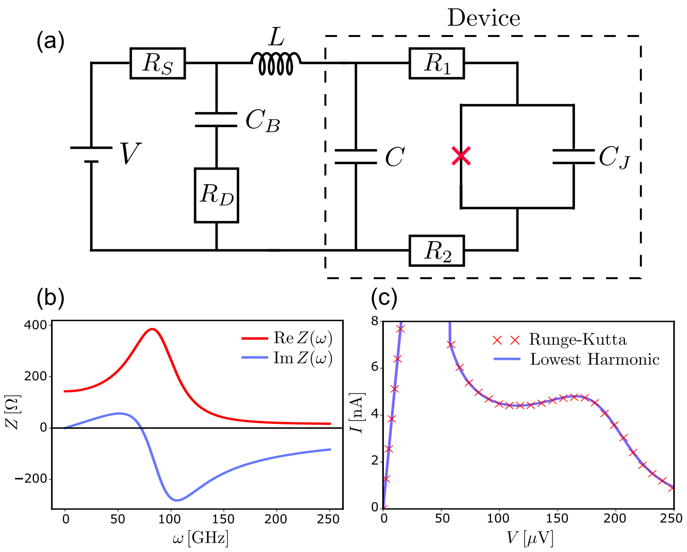

FIG. S5. (a) Lump element circuit used to model the experiment. The dashed section highlights the part of the circuit stemming from the on-chip device itself. (b) Impedance for the circuit in (a) using the fit parameters from the main text: $R_S = 1920\ \Omega$, $R_1 + R_2 = 30\ \Omega$, $R_D = 120\ \Omega$, $C = 6.5$ fF, $C_J = 2.4$ fF, $C_B \to \infty$, $L = 0.34$ nH, $I_c = 64$ nA, $V_{sw} = 0.46 Z(0) I_c$. (c) Theoretical I-V using parameters from (b). 'Runge-Kutta' is obtained via solving Eq. (7) numerically, while 'Lowest Harmonic' comes from Eq. (13). The corresponding Fig. 2(c) in the main text uses a different symbol for the Josephson junction: there the combination of voltage bias and the Josephson junction (x) is replaced by a symbol of an ac voltage source.

capacitance. As an order of magnitude estimate, we set the weakly (current) ramp dependent logarithmic term to a realistic value $\ln(\alpha\tau) \approx 5$. Substituting these numbers in Eq. (2) we obtain $I_s/I_c \approx 0.4$. The switching current is then $I_s \approx 26$ nA which is similar to the value that we measured and presented in Fig. 3(a) in the main text.

V. THEORY AND FITTING DETAILS

In this section we add more detail to the theory utilized to fit the experiment in the main text, and we explain how the fitting is performed. We limit ourselves to a classical description of the circuit, and neglect effects of finite temperature and electrical noise, which we expect to play only a minor role.

As in the main text we describe the experimental circuit via lump elements, with the full circuit shown in Fig. S5(a) with the dashed area indicating elements associated with the on-chip device, with the rest associated to the larger circuit external to the chip. Here C_B, which describes the contacts on the sample stage outside the chip, is assumed to be very large, $C_B \to \infty$, which separates the impedance into a zero-frequency part, $Z(\omega = 0) = R_1 + R_2 + R_S$, and a finite frequency part as

$$Z(\omega) = \left[iC_J\omega + \frac{1}{R_1 + R_2 + Z_{LC}(\omega)} \right]^{-1}, \tag{3}$$

$$Z_{LC}(\omega) = \left[iC\omega + \frac{1}{iL\omega + \frac{R_S R_D}{R_S + R_D}} \right]^{-1}, \tag{4}$$

with $Z(\omega)$ denoting the frequency dependent series impedance experienced by the junction. In these equations R_1 and R_2 denote HEB resistances, R_D the assumed dark resistance, L bonding wire inductance, C_B bonding pad capacitance (assumed to be large), C_J the capacitance of the junction, C the capacitance of the device bonding pads, and R_S the series resistance from the load resistors on the chip and the line and bias resistances from room temperature to the chip. The full impedance is plotted in Fig. S5(b), using the same parameters as the fit in the main text. The main features of this impedance is a LC resonance, $\omega_{LC} \approx \frac{1}{\sqrt{L(C+C_J)}}$, which is broadened mainly by R_D. Below the resonance, $\omega \ll \omega_{LC}$, the real part of the impedance saturates at $Z(\omega) \approx R_1 + R_2 + \frac{R_D R_S}{R_D + R_S}$, while above the resonance the real part goes to zero as all current runs through the capacitance's, $Z(\omega) \approx \frac{-i}{(C_J + C)\omega}$. Next, we turn our attention to the Josephson junction.

The principal equations of a Josephson junction are,

$$I(t) = I_c \sin\varphi(t), \qquad \frac{d\varphi}{dt} = \frac{2e}{\hbar} V_{JJ}(t) \tag{5}$$

with $V_{JJ}(t)$ being the junction voltage drop. Putting the junction in series with $Z(\omega)$ and a source at voltage V yields

$$V = V_{JJ}(\omega) + Z(\omega) I_c \sin\varphi(\omega), \tag{6}$$

$$V = V_{JJ}(t) + \int d\tau Z_t(t-\tau) I_c \sin\varphi(\tau). \tag{7}$$

with the second equation being the Laplace transform of the first and $Z_t(t)$ the Fourier transform of $Z(\omega)$. For $|V| \leq Z(0)I_c$ a trivial solution exists with a constant $\sin\varphi = \frac{V}{I_c Z(0)}$, corresponding to the supercurrent branch. However, since $Z(0) > |Z(\omega)|$ the circuit is underdamped with the possibility of multiple solutions. In a full treatment, finite temperature and noise would render parts of the supercurrent branch unstable with a switching voltage $V_{sw} \leq Z(0)I_c$. Here to simplify, we choose $V_{sw} = 0.46 Z(0) I_c$ to match experiment and only consider solutions for $\varphi \neq$ const. at $|V| > V_{sw}$.

To obtain steady-state solutions after the switching one has to solve Eq. (7). This can be done numerically by Runge-Kutta method, with results shown in Fig. S5(c), or analytically by keeping only the lowest harmonic of the Josephson frequency, $\omega_J/2\pi$, for which the phase is assumed to evolve as,

$$\varphi(t) = \omega_J t + \varphi_A \sin(\omega_J t + \delta), \tag{8}$$

in steady-state. Inserting this into Eq. (7), separating Fourier components, and keeping only the lowest order Bessel terms of $\sin\varphi(t)$, we find the following three equations,

$$V = \frac{\hbar}{2e}\omega_J + Z(0) I_c J_1(\varphi_A) \sin\delta, \tag{9}$$

$$\frac{J_0(\varphi_A)}{\varphi_A} = \frac{\hbar}{2e}\frac{\omega_J}{I_c |Z(\omega_J)|}, \tag{10}$$

$$\delta = -\arctan\left(\frac{\operatorname{Re} Z(\omega_J)}{\operatorname{Im} Z(\omega_J)}\right). \tag{11}$$

To investigate these equations order-by-order in φ_A, we start by assuming $V \simeq \frac{\hbar}{2e}\omega_J$, and further in the voltage range of interest, comprising Regimes 1 and 2 of the main paper, $V = \hat{V} Z(0) I_c$ with $\hat{V} \in \{0.1, 10.0\}$. Now, Eq. (10) can be expressed as,

$$\frac{J_0(\varphi_A)}{\varphi_A} = \hat{V}\frac{Z(0)}{|Z(\omega_J)|} \approx \frac{1}{\varphi_A}, \tag{12}$$

with the last approximation being valid for an underdamped junction, $Z(0) \gg |Z(\omega)|$, since $J_0(x) \leq 1.0$. Next, inserting this result into Eq. (9) we find the deviation from $V = \frac{\hbar}{2e}\omega_J$ to be of order φ_A, meaning that if included in Eq. (10) would generate corrections $\mathcal{O}(\varphi_A^2)$, which are negligible for our parameters. Likewise, if the next harmonic of $\varphi(t)$ at frequency $2\omega_J$ is kept, it would result in terms of order $\sim \varphi_A \frac{|Z(2\omega_J)|}{\hat{V} Z(0)} \propto \varphi_A^2$ and thus likewise neglible. These higher harmonic terms stem from the feedback of the first harmonic, i.e. the junction experiences an ac drive it itself generated, which in turn generates even higher harmonics. This is similar to the physics of Shapiro steps where a constant ac drive yields a ladder of coupled harmonics, although for our case the ac drive is weak, $\mathcal{O}(\varphi_{\mathcal{A}})$, and subsequent harmonics are therefore suppressed.

To linear order in φ_A we find,

$$I_0 = I_c^2 \frac{e}{\hbar}\frac{\operatorname{Re} Z(\omega_J)}{\omega_J}, \qquad V = \frac{\hbar}{2e}\omega_J + Z(0) I_0, \tag{13}$$

with I_0 denoting dc current and matching Eqs. (2) of the main text. These equations can be jointly solved to obtain the I-V, shown as the full line in Fig. S5(c). The perfect correspondence between numerical solutions of Eq. (7) and Eq. (13) supports this expansion. Lastly, we comment that an identical result to Eq. (13) can be obtained from $P(E)$ theory by expanding to lowest order in $\operatorname{Re} Z(\omega)/R_K$, with $R_K = \frac{h}{2e^2}$ denoting the resistance quantum, highlighting that the classical limit is justified [5, 6]. To obtain the dc power deposited in HEBs we use $P_j = R_j \langle I_j^2 \rangle_0$, with I_j

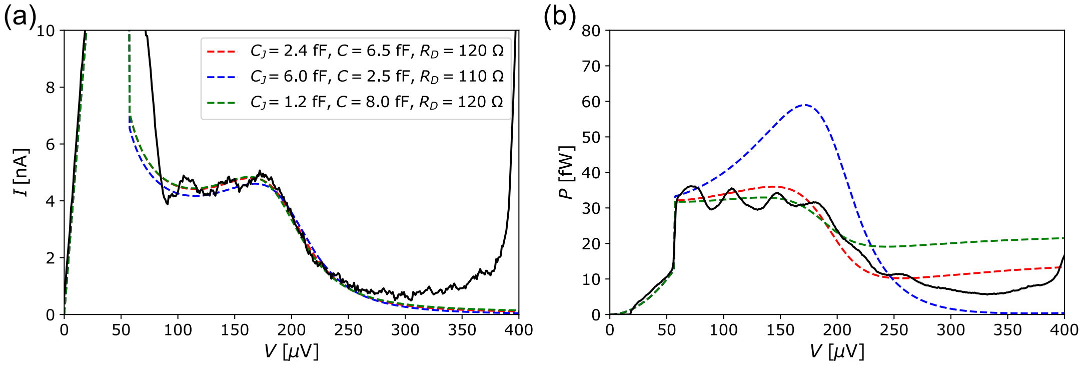

FIG. S6. (a) I-V's fitted to experiment for different values of C_J, C and R_D, with remaining parameters matching those in Fig. S5(b). (b) Power absorbed in R_1 for the three parameter sets in (a), highlighting that the HEB measurement can determine the C/C_J scale.

being the current through HEB j, and index 0 indicating dc component. The full current and voltage drop across the junction is given by

$$V_{JJ}(t) = \frac{\hbar}{2e}\omega_J - I_c \mathrm{Re}\, Z(\omega_J)\sin(\omega_J t) - I_c \mathrm{Im}\, Z(\omega_J)\cos(\omega_J t), \tag{14}$$

$$I(t) = I_0 + I_c \sin(\omega_J t), \tag{15}$$

using Eqs. (13), the second Josephson relation, and by expanding the sine in harmonics in Eq. (5). The current through a HEB is then given by,

$$I_j(t) = I(t) - C_J \frac{dV_{JJ}}{dt} \tag{16}$$

which is used to obtain Eq. (3) of the main text. For $\omega_J \gg \omega_{LC}$ all current runs through the capacitors and $I_j(t) \approx \frac{I_c}{1+C_J/C}\sin\omega_J t$, hightlighting that the ratio C_J/C controls the distribution of power in this regime. In general, the measurement of power allows us to determine the capacitive and inductive terms more precisely. This is highlighted in Fig. S6 where we show multiple fits for the I-V's of the main text. Here, the I-V's are of almost equal quality, but plotting power for the same parameters reveals differences.

Next, we turn to the fitting of circuit parameters used in the main paper. First, the resistance of the thermometers is estimated to be $R_1 + R_2 = 30\ \Omega$, and then from matching the supercurrent branch we find $R_S = 1920\ \Omega$. Next, there are three main features we utilize to extract parameters; 1) to fit the experimental I-V we require an LC frequency $\omega_{LC}/2\pi \approx 93$ GHz and $R_D \approx 120\ \Omega$ to place and broaden the resonance correspondingly, 2) to match the drop in power in Regime 3 we require a fixed C/C_J ratio, 3) to fit the scaling between $\mathrm{Re}\,(Z(\omega))$ amplitude and resonance width the RL to $\sqrt{CL}$ scale needs to be fixed. Fulfilling theses conditions we find a fit of $C_J = 2.4$ fF, $C = 6.5$ fF and $L = 0.34$ nH, which captures both the I-V and the power signal. Geometric estimates place $C_J \approx 5$ fF and $C \approx 12$ fF, which are quite consistent with the fit values. The simple lump element approximations, and the assumption of $R_D = const.$ in frequency imply that we do not expect full quantitative coincidence between the simulation and geometric expectations. With no prior knowledge of the origin of R_D, which could relate to either inductive loss, capacitive loss, or other finite frequency losses, we elect to remain within our minimal description.

Next, we discuss the distribution of total applied power, $P = I_0 V$, in the circuit in Regimes 2 and 3 of the main text. The power captured by the HEBs are given by Eq. (3), while the line resistors, R_S, capture only the dc component, $P_S = R_S I_0^2$, and lastly the power absorbed by R_D follows from exclusion $P_D = P - P_S - P_1 - P_2$. The results of these estimates are shown in Table I. Here it is seen that for $V = 90\ \mu$V the HEBs absorb around $\sim 20\%$ of the total power, while for $V = 170\ \mu$V and $V = 250$ this ratio is closer to 10%. To note; this assumes that all power is absorbed in the form of currents running through resistance, while other sources might also contribute e.g. excited quasiparticles absorbed near the junction in Regime 4.

Lastly, we highlight that the ratio of R_D to R_j determines the ratio of power absorbed by the HEBs with respect to full power. The value of $R_D = 120\ \Omega$, found by fitting I-V, fits well with the experimental estimates of $\sim 10-20\%$. In Fig. S7 we show how the effect of increasing resistances R_j would increase the power captured by the HEBs, while largely retaining the shape of the I-V. Consequently, a larger ratio than 20% is captured by HEBs in this scenario. However, for a large increase of R_j one approaches the breakdown of the underdamped regime and higher Fourier components might further complicate the dynamics.

	P (f W)	P_S (f W)	P_1 (f W)	P_2 (f W)	P_D (f W)
$V = 90\ \mu$V	360	30	35	35	260
$V = 170\ \mu$V	750	40	30	30	650
$V = 250\ \mu$V	250	2	10	10	228

TABLE I. Table of estimated power distribution in the circuit in Regimes 2 and 3 of the main text.

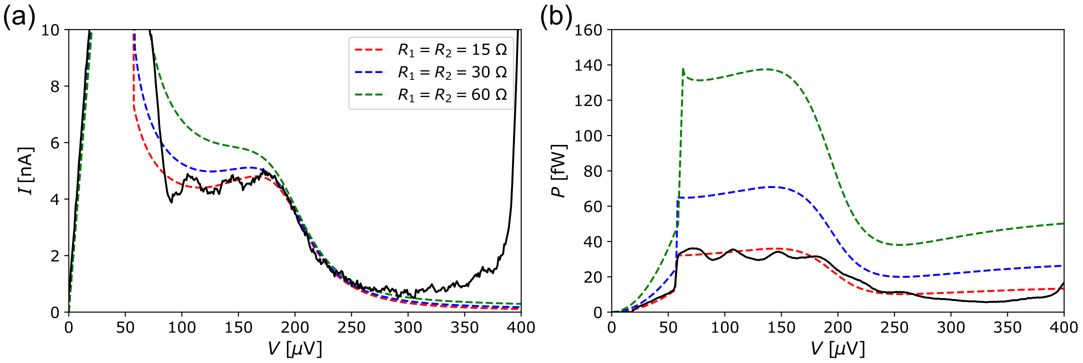

FIG. S7. (a) I-V's for different values of R_1, R_2, with remaining parameters matching those in Fig. S5(b). (b) Power absorbed in R_1 for the three parameter sets in (a), illustrating that the HEBs capture more of the ac power at higher resistance R_j. Note that for $R_1 = R_2 = 60\,\Omega$ the utilized underdamped assumption is invalid for low voltages, rendering the curve only approximate around $V \sim 50 - 100\ \mu$V.

[1] J. M. Kivioja, T. E. Nieminen, J. Claudon, O. Buisson, F. W. J. Hekking, and J. P. Pekola, Observation of Transition from Escape Dynamics to Underdamped Phase Diffusion in a Josephson Junction, Phys. Rev. Lett. **94**, 247002 (2005).
[2] A. I. Larkin and Yu. N. Ovchinnikov, Zh. Eksp. Teor. Fiz. **91**, 318 (1986); [Sov. Phys. JETP 64, 185 (1987)]. A. I. Larkin and Yu. N. Ovchinnikov, Zh. Eksp. Teor. Fiz. **87**, 1842 (1984); [Sov. Phys. JETP 60, 1060 (1984)].
[3] J. P. Pekola, K. P. Hirvi, J. P. Kauppinen, and M. A. Paalanen, Thermometry by Arrays of Tunnel Junctions, Phys. Rev. Lett. **73**, 2903 (1994).
[4] Jukka P. Pekola and Bayan Karimi, Colloquium: Quantum heat transport in condensed matter systems, Rev. Mod. Phys. **93**, 041001 (2021).
[5] Gert-Ludwig Ingold and Yu. V. Nazarov, Charge Tunneling Rates in Ultrasmall Junctions, arXiv.cond-mat/0508728
[6] Landry Bretheau, Localized Excitations in Superconducting Atomic Contacts: PROBING THE ANDREEV DOUBLET. PhD thesis, Ecole Polytechnique X, February 2013.
