# Supplementary material for: Bolometric detection of Josephson radiation
Source: Nat Nanotechnol. 2024 Aug 22;19(11):1613–8. doi: 10.1038/s41565-024-01770-7 (PMC11567893; doi:10.1038/s41565-024-01770-7)
Supplement: Supplementary file 2 — Original SEM images of the measured device, with no colour and full size. [file 41565_2024_1770_MOESM2_ESM.pdf]

Fi. 2(b)
top, main

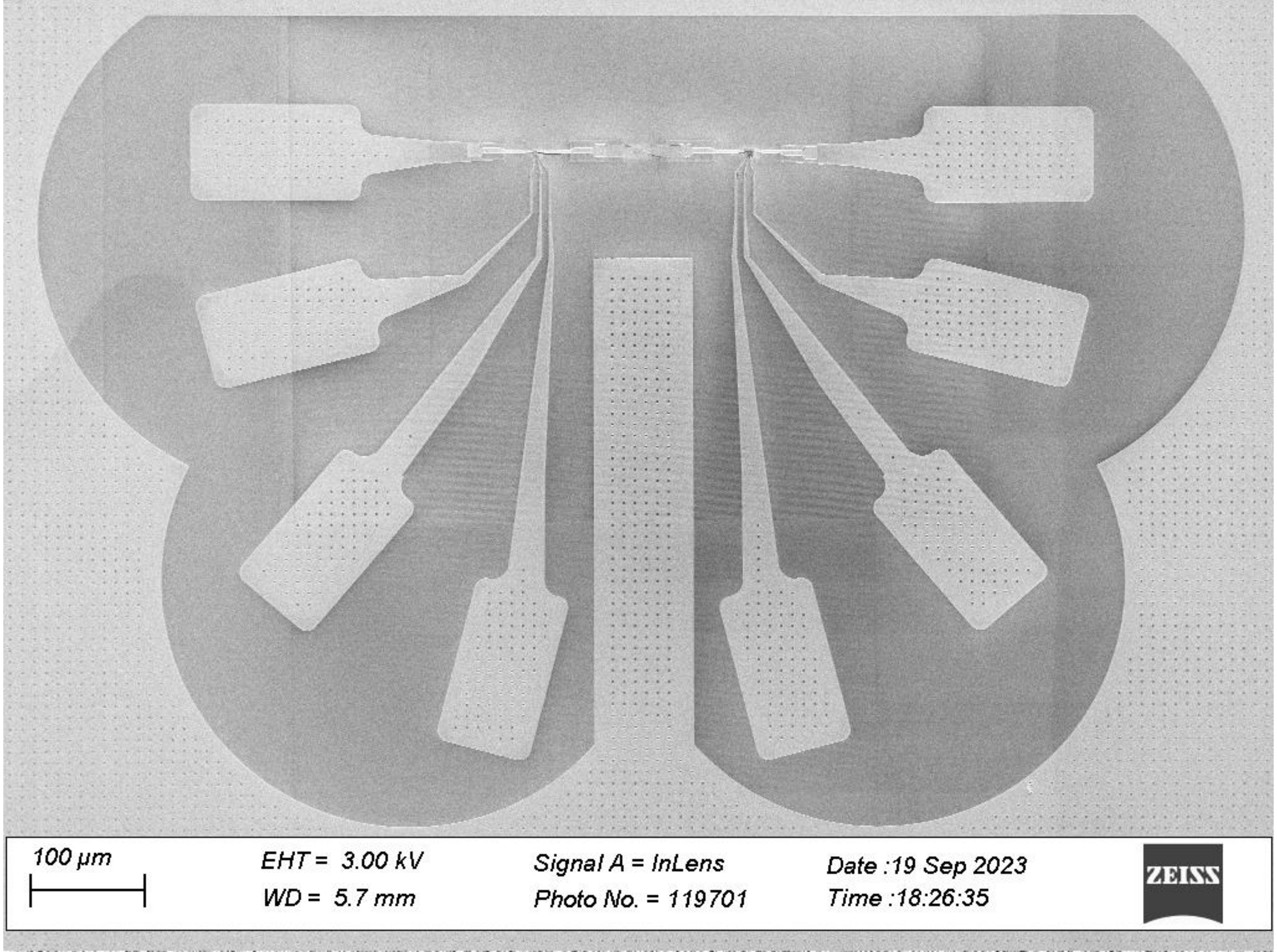

Fi. 2(b)
bottom, main

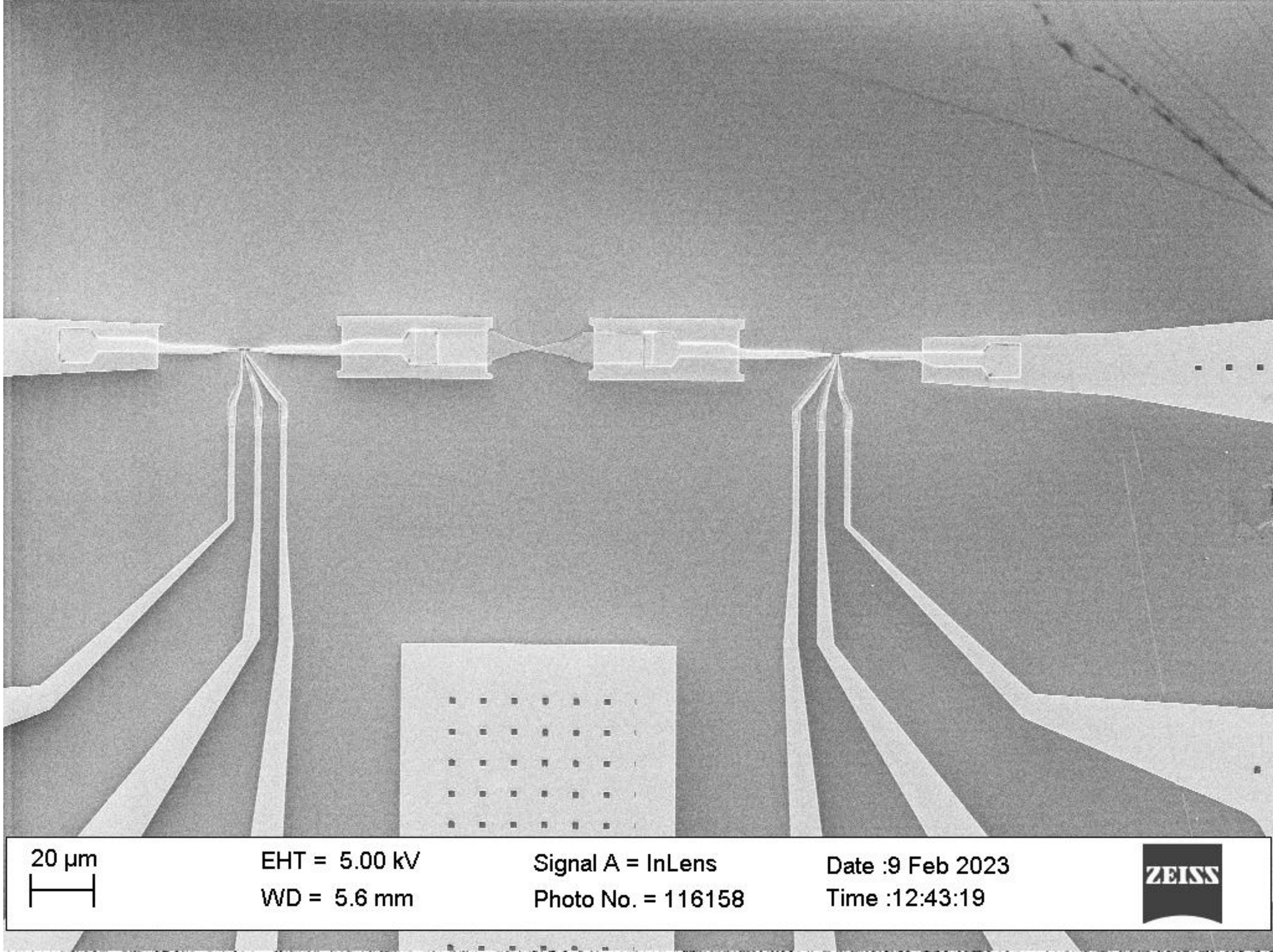

Fi. 2(b)
bottom,
Left inset

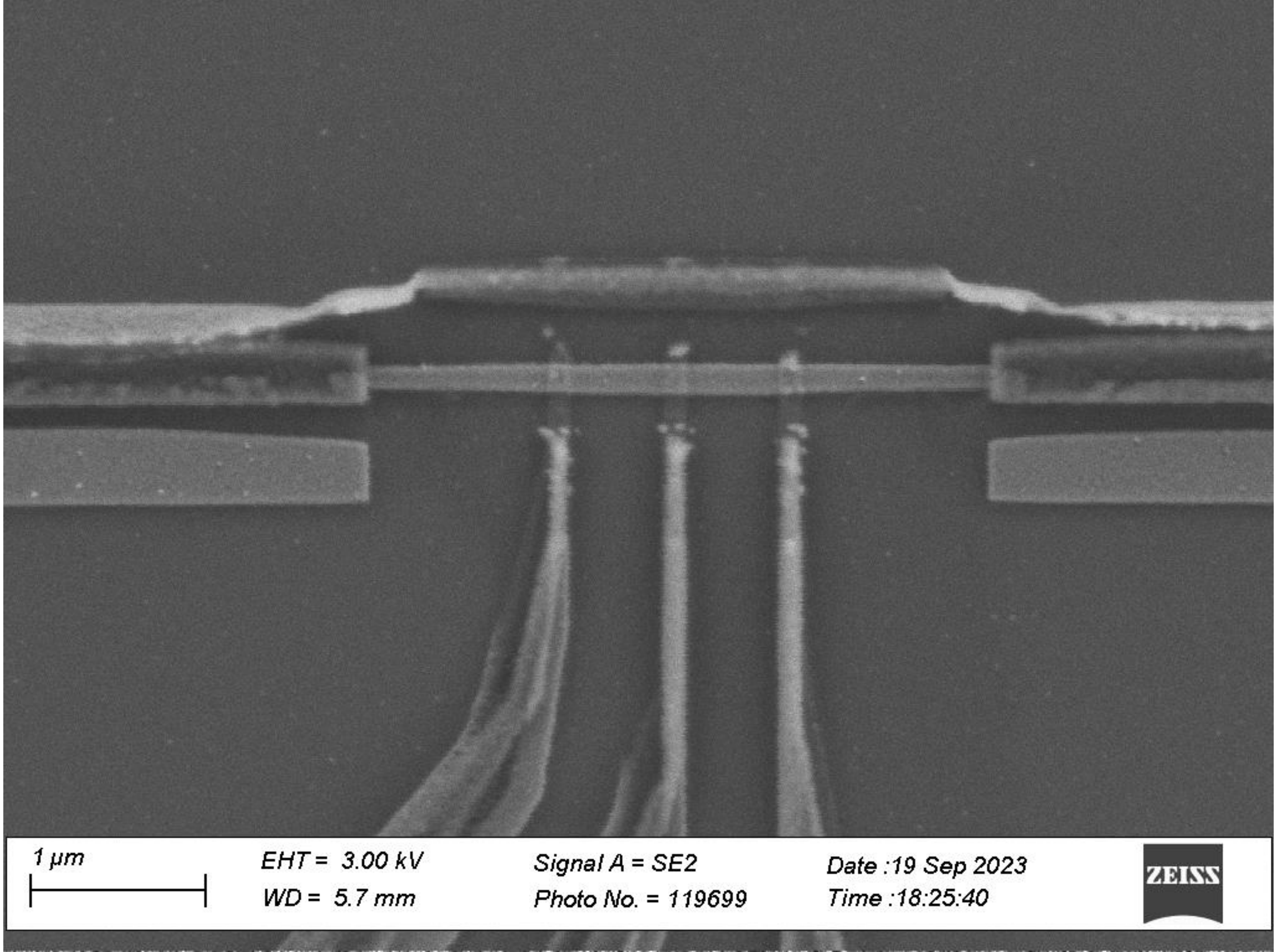

Fi. 2(b)
bottom,
right inset

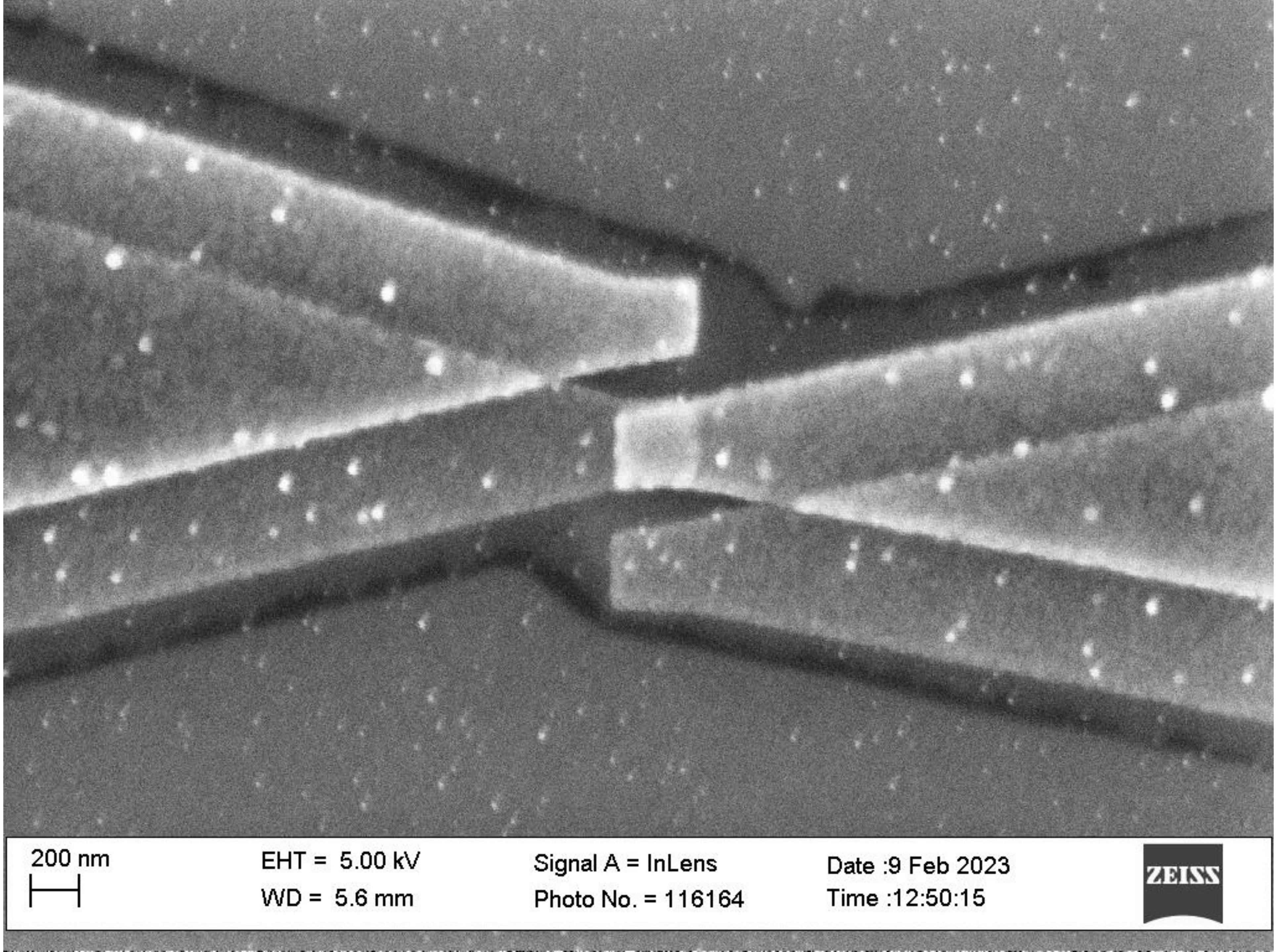

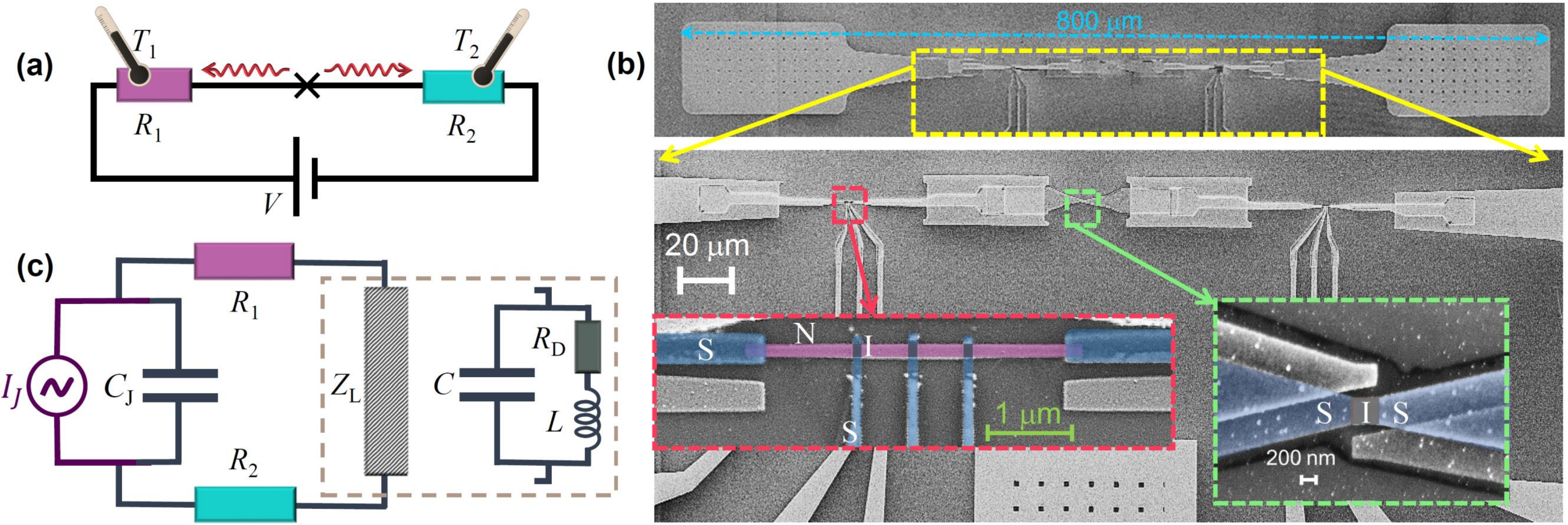
(a)
T_1
T_2
R_1
R_2
V
(b)
800 μm
20 μm
S
N
I
S
1 μm
S
I
S
200 nm
(c)
R_1
I_J
C_J
R_2
Z_L
C
R_D
L
